# In-Vitro Measurement of Forces During Debridement with a Piezoelectric Ultrasonic Periodontal Scaler

**DOI:** 10.3290/j.ohpd.b5458595

**Published:** 2024-06-12

**Authors:** Diego Stutzer, Martin Hofmann, Sigrun Eick, Nicole Scharp, Jürgen Burger, Thomas Niederhauser

**Affiliations:** a PhD Student, Institute for Human Centered Engineering, Bern University of Applied Sciences, Biel, Switzerland. Conceptualisation, data curation, formal analysis, investigation, methodology, resources, software, validation, visualization, wrote, reviewed and edited the manuscript.; b Head of Ultrasonics in Medicine and Engineering, School of Biomedical and Precision Engineering, University of Bern, Bern, Switzerland. Investigation, resources, reviewed and edited the manuscript.; c Professor and Head of Laboratory for Oral MicrobiologyDepartment of Periodontology, School of Dental Medicine, University of Bern, Switzerland. Funding acquisition, investigation, resources, reviewed and edited the manuscript.; d Head of Clinical Education, PET College for Dental Hygiene, Careum Educational Center, Zürich, Switzerland. Funding acquisition, investigation, resources, reviewed and edited the manuscript.; e Professor for Precision Engineering and Translational Medicine, Directorate of Studies, School of Biomedical and Precision Engineering, University of Bern, Bern, Switzerland. Funding acquisition, supervision, reviewed and edited the manuscript.; f Professor for Biomedical Signal Processing and Control, Head of Institute for Human Centered Engineering HuCE, Bern University of Applied Sciences, Biel, Switzerland. Funding acquisition, supervision, reviewed and edited the manuscript.

**Keywords:** calculus, debridement, periodontal, piezoelectric, ultrasonic

## Abstract

**Purpose:**

This study investigated the magnitude, direction, and temporal aspects of the force applied during instrumentation with a piezoelectric ultrasonic periodontal scaler, compared this force with recommendations in the literature, and assessed the influence of the profession (dentist or dental hygienist) and calculus hardness.

**Materials and Methods:**

The force applied by ten dental hygienists and six dentists during debridement of comparatively soft and hard artificial dental calculus with a piezoelectric ultrasonic scaler was recorded in-vitro. The total force and its components in three axes were statistically analysed.

**Results:**

During debridement of soft artificial dental calculus, the mean total force applied by dental hygienists was 0.34 N (± 0.18 N, range: 0.13 N to 0.59 N) and by dentists 0.28 N (± 0.33 N, range: 0.06 N to 0.95 N), and the total force exceeded 0.5 N approximately 23% and 14% of the time for dental hygienists and dentists, respectively. During debridement of hard artificial dental calculus, the mean total force applied by dental hygienists was 0.63 N (± 0.40 N, range: 0.28 N to 1.64 N) and by dentists 0.57 N (± 0.17 N, range: 0.34 N to 0.76 N); the total force exceeded 0.5 N more than half of the time for both professions. On average, dental hygienists applied 1.85x (p = 0.04) and dentists 2.04x (p = 0.06) higher force on hard than on soft artificial calculus. However, dental hygienists and dentists used similar forces during the debridement of both hard (p = 1.00) and soft (p = 0.26) calculus.

**Conclusion:**

The force applied during the debridement of hard artificial dental calculus was statistically significantly higher than during the debridement of soft artificial dental calculus. No statistically significant difference between dentists and dental hygienists was found. The force applied by both groups on soft and hard artificial dental calculus frequently exceeded recommended values.

Periodontitis can seriously impair the quality of life and affects millions of persons worldwide.^11^ Treatment typically includes instrumentation by professionals, i.e., a dentist, dental hygienist, or periodontist, to remove dental calculus and biofilm.^13^ While manual instrumentation is still often used, power-driven scalers have become popular in high-income countries. On the one hand, ultrasonic debridement may improve the removal of dental calculus and biofilm while protecting the gingiva and teeth. On the other hand, ultrasonic instruments reduce the tactile sensation.^1^ Furthermore, several studies indicate that ultrasonic debridement may result in higher surface roughness^5,7^ and tooth-substance loss.^14^ However, opposite results have also been reported.^2,6,19^ Various studies have shown that the extent of detrimental effects correlates with the magnitude of force applied during ultrasonic debridement.^1,3-5,7,12,14,18^ Particularly, Flemming et al^3^ recommend limiting the lateral force to 0.5 N to prevent damage. Furthermore, an increased force may reduce the amplitude of the tip’s oscillation and consequently reduce the instrument’s efficiency.^17^

In a pilot study, Ruppert et al^13^ measured the force applied by ten dentists and ten dental hygienists during debridement of a bicuspid with a magnetostrictive ultrasonic scaler (Cavitron SPS, Dentsply; Konstanz, Germany). They found that the mean positive force applied by dental hygienists and dentists was 0.77 N and 1 N, respectively.^13^

The force applied using contemporary piezoelectric ultrasonic scalers may differ from the force applied using magnetostrictive ultrasonic scalers, since piezoelectric scalers feature a different shape of the instrument tip and housing, different vibration intensity, and arguably a less elliptical pattern of the tip’s motion.^8-10,15^

Moreover, Ruppert et al^13^ identified that the professionals involved in their study might need more training and guidance on the use of the magnetostrictive scaler. However, to the authors’ best knowledge, no data is available regarding the force applied by trained professionals during the use of contemporary piezoelectric ultrasonic scalers. Furthermore, there is a lack of information on the direction of the applied force with respect to the treated surface as well as on temporal aspects, such as the temporal distribution and the rate of change of the applied force.

The goal of the present study was to quantify the force applied by professionals during debridement of comparatively soft and hard artificial calculus with a contemporary piezoelectric ultrasonic scaler and to assess the influence of profession and calculus hardness. The present study investigated the magnitude, direction, and temporal aspects of the force applied during debridement with a piezoelectric ultrasonic scaler in an in-vitro setup. The resulting data may help to raise awareness about the risk of unintentionally applying excessive and potentially detrimental force. Furthermore, the results can help to optimise the performance of piezoelectric scalers for clinical use.

## Materials and Methods

### Measurement Setup

**Fig 1:**
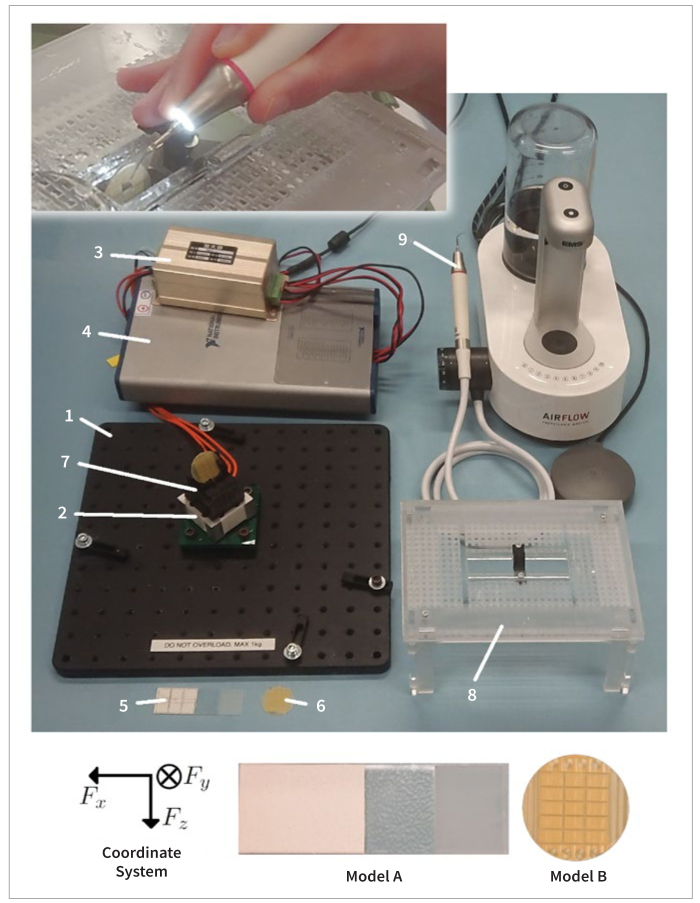
Image taken during debridement (top left) using the measurement set-up (center): base plate (1); three-axis force sensor (2) with amplifier (3); data acquisition module (4); Model A (5) with comparatively soft and Model B (6) with comparatively hard artificial dental calculus; adapters (7), handrest (8); and piezoelectric ultrasonic scaler (9) and definition of coordinate system aligned to Model A and Model B (bottom).

To record the force applied by the professionals during debridement, a custom-built measurement system was used (Fig 1). The measurement system comprises a base plate (1), a three-axis force sensor (ZM3DW-AL 10N, Anhui Zhimin El. Tech.; Bengbu, China) (2), including an amplifier (3), a data acquisition module (USB-6366, National Instruments; Austin, TX, USA) (4), Model A (5) with comparatively soft and Model B (6) with comparatively hard calculus, custom-built adapters to mount calculus models on the force sensor (7), a custom-built handrest (8), a piezoelectric ultrasonic periodontal scaler (PIEZON EN-060 LED Handpiece with PIEZON PS Instrument, Electro Medical Systems; Nyon, Switzerland) (9), and a computer and video camera not shown in Fig 1.

The force sensor was mounted on the base plate and connected to the computer via the data acquisition module. A given calculus model was mounted on the force sensor using the dedicated adapter. The handrest was mounted on the base plate to provide adequate support for the fingers during debridement. The computer was used to record the force applied by the professionals, and the video camera was used to record the position of the scaler during the debridement.

Model A comprised a thin layer (~0.1 mm) of comparatively soft artificial dental calculus with a mean hardness of 4.6 HV (± 0.8 HV) made of a mixture of 10 weight-parts of ‘‘Miocolor Aqua Hartgrund Farblos’’ (Migros Genossenschafts Bund; Zürich, Switzerland), and 6 weight-parts ‘‘Krone Gips’’ (Hilliges Gipswerk; Osterode am Harz, Germany), on a rectangular glass slab.^16^

Model B comprised a thin layer (~0.1 mm) of comparatively hard artificial dental calculus with a mean hardness of 24 HV (± 4.9 HV) made of a mixture of 10 weight-parts of ‘‘Neukadur Multicast 15’’, 23 weight-parts of ‘‘Neukadur Härter ISO3’’ (Altropol Kunststoff; Stockelsdorf, Germany), and 33 weight-parts ‘‘Omyacarb 30 µm’’ (Omya; Oftrigen, Switzerland), on a circular glass plate.^16^

The three-axis force sensor was calibrated by the manufacturer. Additionally, the validity of the force measured along all three axes was verified before and after each set of measurements using a calibrated spring scale (Medio-Line 40010, Pesola Präzisionswaagen; Chur, Switzerland).

### Measurement Procedure

Based on the results by Ruppert et al^13^ and discussions with therapists and the manufacturer of the ultrasonic periodontal scaler, we expected that therapists would apply a force of approximately 0.25 N ± 0.1 N. Assuming that a difference of 0.25 N is clinically relevant, the power of a study with five samples to detect this difference using a two-sample pooled t-test would be 0.93.

Finally, ten dental hygienists and six dentists were invited to debride Model A and Model B. Before each measurement, the professionals were queried to ensure they were familiar and experienced with the piezoelectric ultrasonic scaler used in this study. All participants reported many years of experience in ultrasonic debridement and with the given ultrasonic scaler. Prior to instrumentation, they were asked to familiarise themselves with the measurement set-up and adjust the scaler’s vibration intensity and water irrigation to settings they considered appropriate for the given calculus model. Subsequently, they were asked to remove artificial dental calculus from a predefined area of 10 x 10 mm^2^ on Model A and 3.5 x 6.5 mm^2^ on Model B. During debridement, the applied force and the scaler’s position were recorded. After the experiments, the professionals were asked to provide feedback about the measurement set-up, the calculus models, and the applied forces.

### Data Processing and Statistical Analysis

The data from the force sensor was processed using MATLAB (R2021a, The MathWorks; Natick, MA, USA). First, the raw data of each measurement was filtered, and the offset of the sensor output of each axis was subtracted. Second, the time frame during which the scaler was in contact with the calculus model was isolated, and the recorded values were converted to a force in a coordinate system aligned with the calculus model (Fig 1). Subsequently, the mean, median, maximum, and distribution of the total force (F_tot_=√[Fx^2^ + Fy^2^ + Fz^2^]) and its components (Fx, Fy, and Fz) were calculated for each measurement.

Finally, statistical values for each combination of profession and model type were calculated from the means, medians, and maximums of individual measurements, as listed in Table 1. Wilcoxon rank-sum tests were used to assess the difference between dental hygienists and dentists and between Model A and Model B using MATLAB’s Statistics and Machine Learning Toolbox v. 9.4 (R2021a, The MathWorks). Moreover, the distributions of the total force and its rate of change were grouped by profession and model type and averaged, resulting in the overall distributions illustrated in Figs 4 and 5.

**Table 1:** Study statistics of mean, median, and maximum values of individual measurements of the total force, its components and its rate of change during debridement of Model A and Model B by dental hygienists and dentists

	Dental hygienists (n=10)						Dentists (n=6)						p-value*
	Mean	SD	Median	IQR	Min	Max	Mean	SD	Median	IQR	Min	Max	
Model A:													
Mean magnitude of F_tot_ [N]	0.34	0.18	0.29	0.38	0.13	0.59	0.28	0.33	0.16	0.07	0.06	0.95	0.26‡
Median magnitude of F_tot_ [N]	0.35	0.18	0.30	0.37	0.13	0.59	0.28	0.34	0.16	0.08	0.06	0.96	0.22‡
Max magnitude of F_tot_ [N]	0.66	0.36	0.57	0.45	0.25	1.47	0.60	0.62	0.40	0.18	0.14	1.83	0.31‡
Mean magnitude of Fx [N]	0.06	0.13	0.01	0.02	0.00	0.41	0.02	0.02	0.01	0.01	0.00	0.05	0.79‡
Mean magnitude of Fy [N]	0.31	0.16	0.29	0.29	0.12	0.56	0.27	0.33	0.15	0.07	0.06	0.94	0.18‡
Mean magnitude of Fz [N]	0.04	0.05	0.02	0.02	0.01	0.17	0.03	0.02	0.03	0.05	0.00	0.06	1.00‡
Mean rate of change of F_tot_ [N/s]	1.34	0.87	1.06	0.40	0.69	3.7	2.16	0.55	2.14	0.48	1.60	3.16	0.01‡
Max rate of change of F_tot_ [N/s]	11.95	9.18	9.59	3.00	5.62	36.77	15.12	4.38	14.58	6.33	8.61	20.37	0.09‡
Model B:													
Mean magnitude of F_tot_ [N]	0.63	0.40	0.56	0.42	0.28	1.64	0.57	0.17	0.60	0.30	0.34	0.76	1.00‡
Median magnitude of F_tot_ [N]	0.61	0.43	0.55	0.43	0.14	1.68	0.55	0.16	0.59	0.28	0.34	0.73	0.96‡
Max magnitude of F_tot_ [N]	1.53	0.38	1.66	0.45	0.91	2.19	1.64	0.50	1.64	1.03	1.03	2.22	0.64‡
Mean magnitude of Fx [N]	0.24	0.33	0.14	0.09	0.06	1.18	0.14	0.06	0.13	0.10	0.08	0.22	0.96‡
Mean magnitude of Fy [N]	0.44	0.19	0.43	0.26	0.19	0.73	0.51	0.17	0.54	0.27	0.29	0.70	0.56‡
Mean magnitude of Fz [N]	0.22	0.30	0.11	0.12	0.06	1.07	0.14	0.06	0.11	0.09	0.10	0.23	0.49‡
Mean rate of change of F_tot_ [N/s]	2.00	0.65	1.82	0.80	1.14	3.37	2.19	0.26	2.16	0.47	1.86	2.51	0.37‡
Max rate of change of F_tot_ [N/s]	29.74	11.69	24.80	17.60	18.18	49.73	39.55	13.67	39.35	14.99	22.27	61.75	0.22‡
p-value† (mean magnitude of F_tot_)	0.03‡						0.09‡						
p-value† (median magnitude of F_tot_)	0.11‡						0.06‡						

* Dental hygienists vs dentists. † Model A vs Model B. ‡ Wilcoxon rank-sum test. F_tot_: total force; N: Newtons.

## Results

**Fig 2:**
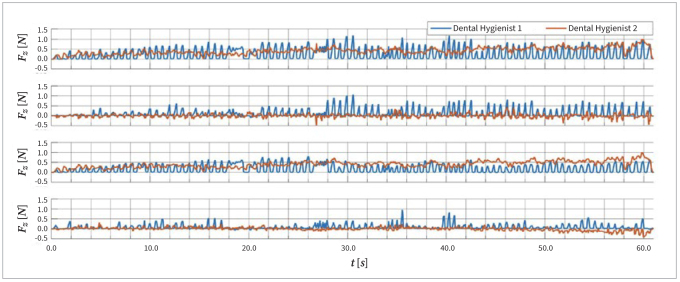
Sections of the total force (F_tot_) and its components (Fx, Fy, and Fz) measured during the debridement of Model B by two dental hygienists exemplarily illustrating two different working patterns.

Figure 2 shows examples of the time series of the total force applied by two different dental hygienists during debridement of Model A, as well as the force’s components with respect to the calculus model surface. For Dental Hygienist 1, all components and the total force (F_tot_) were basically fluctuating between ~0 N and positive values. In contrast, Dental Hygienist 2 applied a working pattern in which the F_tot_ and its component perpendicular to the model surface (Fy) continuously remained positive throughout the entire debridement, while the components parallel to the model surface (Fx and Fz) oscillated between positive and negative values.

Table 1 depicts the study’s overall statistics for each combination of profession and calculus model.

During debridement of Model A, the mean total force ranged from 0.13 N to 0.59 N for dental hygienists, resulting in an overall mean of 0.34 N and a SD of 0.18 N, and from 0.06 N to 0.95 N for dentists, resulting in an overall mean of 0.28 N (± 0.33 N). The average maximum total force was 0.66 N (± 0.36 N) for dental hygienists and 0.60 N (± 0.62 N) for dentists, and forces up to 1.83 N were recorded. The mean rate of change ranged from 0.69 N/s to 3.70 N/s, resulting in an overall mean of 1.31 N/s (± 0.87 N/s) for dental hygienists and from 1.60 N/s to 3.16 N/s, resulting in an overall mean of 2.16 N/s (± 0.55 N/s) for dentists.

During debridement of Model B, the mean total force ranged from 0.28 N to 1.64 N for dental hygienists, resulting in an overall mean of 0.63 N (± 0.40 N), and from 0.34 N to 0.76 N for dentists, resulting in an overall mean of 0.57 N (± 0.17 N). The average maximal total force was 1.53 N (± 0.38 N) for dental hygienists and 1.64 N (± 0.50 N) for dentists, and forces up to 2.22 N were recorded. The mean rate of change ranged from 1.14 N/s to 3.37 N/s, resulting in an overall mean of 2.00 N/s (± 0.65 N/s) for dental hygienists, and from 1.86 N/s to 2.51 N/s, resulting in an overall mean of 2.19 N/s (± 0.26 N/s) for dentists.

In comparison, the mean total force applied on hard artificial calculus was 1.85x (p = 0.04) and 2.04x (p = 0.06) higher times higher than on soft artificial calculus for dental hygienists and dentists, respectively.

Comparing the two professions, the mean total force applied by dental hygienists was 1.21x (p = 0.26) and 1.11x (p = 1.00) higher than the force applied by dentists on soft and hard artificial calculus, respectively.

The distribution of the medians is similar to the distribution of the means, as can be seen in Table 1.

**Fig 3:**
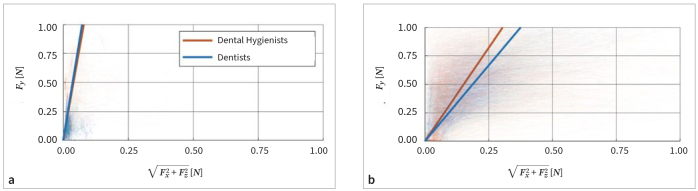
Scatter plots and linear regressions of the force component parallel (√[Fx^2^ + Fz^2^]) and perpendicular (Fy) to the surface of the calculus model measured during debridement of Model A (a) and Model B (b) by dental hygienists and dentists.

The scatter plots and simple linear regressions in Fig 3 illustrate the relation between the component of the force parallel to the calculus model surface (√[Fx^2^+Fz^2^]) and the component perpendicular to the surface (Fy). During debridement of Model A, the force component perpendicular to the surface was, on average, about 12.6x and 13.7x larger than the component parallel to the surface for dental hygienists and dentists, respectively. During debridement of Model B, the perpendicular component was about 3.3x and 2.7x larger than the parallel component for dental hygienists and dentists, respectively.

**Fig 4:**
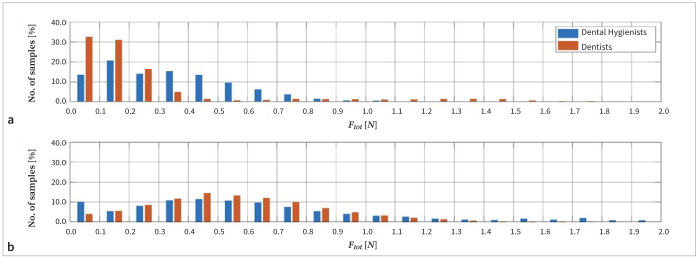
Average distributions of total force (F_tot_) applied by dentists and dental hygienists during debridement of Model A (a) and Model B (b). The relative number of instances of forces exceeding 2 N is not shown.

Figure 4 illustrates the average frequency distributions of the magnitude of the total force. During the debridement of Model A by dental hygienists and dentists, the total force exceeded 0.5 N approximately 23% and 14% of the time, and during debridement of Model B, 54% and 56% of the time, respectively. The lateral force is distributed similarly, and exceeded 0.5 N approximately 20% and 14% of the time during debirdement of Model A by dental hygienists and dentists, and 42% and 48% of the time during debirdement of Model B, respectively.

**Fig 5:**
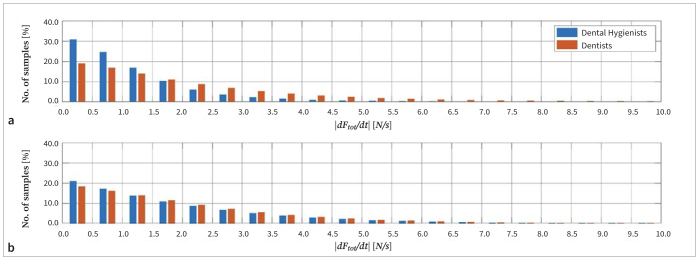
Average distributions of the rate of change of the total force (|dF_tot_/dt|) applied by dental hygienists and dentists during debridement of Model A (a) and Model B (b). The relative number of changes exceeding 10 N/s is not shown.

Figure 5 illustrates the average frequency distributions of the rate of change of total force magnitude. On average, the total force changed faster than 2.0 N/s during debridement of Model A ~17% of the time for dental hygienists, and ~39% of the time for dentists, and during debridement of Model B, ~37% of the time for the dental hygienists, and ~40% of the time for the dentists. Values up to 61.8 N/s were recorded, and the average maximal rate of change during debridement of Model A was 11.9 N/s and 15.1 N/s for dental hygienists and dentists, respectively, and during debridement of Model B, 29.7 N/s and 39.5 N/s, respectively.

## Discussion

This is the first rigorous study to investigate and quantify the magnitude and direction of forces applied during instrumentation with a contemporary piezoelectric ultrasonic periodontal scaler. A custom-made in-vitro measurement set-up was used to record the forces applied by ten dental hygienists and six dentists during debridement of relatively soft (Model A) and comparatively hard (Model B) artificial dental calculus.

The results indicate that the applied force varies widely during individual treatments and between professionals.

On average, the mean force applied by the dental hygienists during debridement was 0.34 N for comparatively soft and 0.63 N for hard artificial dental calculus. The dentists, on average, applied a mean force of 0.28 N on comparatively soft and 0.57 N on hard artificial dental calculus. No statistically significant difference was found between the force applied by dental hygienists and dentists. In contrast, the force applied during the debridement of comparatively soft artificial dental calculus was statistically significantly lower than during the debridement of comparatively hard artificial dental calculus. We suspect that therapists intuitively increase the applied force when the debridement progresses slowly, and an increased force increases the debridement speed.

The mean forces measured in this study are lower than those reported by Ruppert et al^13^ during in-vivo debridement with a magnetostrictive ultrasonic scaler. With the magnetostrictive scaler, a mean positive force of 0.77 N was measured for dental hygienists and 1.00 N for dentists.^13^ The in-vitro measurement set-up used in this study may have contributed to this difference, as it provides facilitated access and a flat surface. In inquiries, the professionals consistently reported that they thus expect marginally lower forces in the in-vitro set-up than in-vivo. In addition, a higher performance of the piezoelectric scaler compared to the magnetostrictive scaler may have contributed to the difference to the results reported by Ruppert et al.^13^ In cases where the debridement progresses faster, the professionals may be less inclined to increase the applied force to speed up the debridement. Furthermore, the study by Ruppert et al^13^ noted that the professionals would need training and guidelines to use the magnetostrictive scaler correctly. In contrast, all dental hygienists and dentists involved in this study were well-trained and experienced in ultrasonic debridement with the given device and were aware of corresponding guidelines. Consequently, we suppose that the lower forces recorded in this study mainly result from a higher level of training and experience of the professionals involved.

Similar to the study by Ruppert et al,^13^ the forces measured in this study were higher than expected. We expected the applied force to generally remain considerably below 0.5 N, as recommended in the literature.^3^ However, higher forces were recorded frequently during the debridement of soft and hard artificial dental calculus. During the debridement of hard artificial calculus, even the applied mean total force was higher than 0.5 N. We consequently suspect that even trained professionals may not be able to estimate or control the magnitude of the applied force during debridement with sufficient accuracy.

Furthermore, the results demonstrate that the force is predominantly applied in a direction perpendicular to the calculus surface. During the measurements, the perpendicular force was 2.7 to 13.7 times larger than the force parallel to the surface. This relationship is to be expected. As recommended by the manufacturer and the literature,^4,5,12,14^ the piezoelectric ultrasonic scaler was mostly held and moved nearly parallel to the calculus surface (Fig 1). In this orientation, the instrument vibrates parallel to the surface and facilitates debridement in the direction of the ultrasonic vibration.

The average ratio between perpendicular and parallel forces was considerably lower during debridement of hard calculus on Model B (3.3 and 2.7) than during debridement of soft calculus on Model A (12.6 and 13.7). This may indicate that the professionals were intuitively aware that the perpendicular force during debridement of the hard calculus could be relatively high. Consequently, they tried to avoid increasing the perpendicular force while applying a higher parallel force to increase the debridement speed.

As illustrated in Fig 2, the work patterns varied considerably between individual professionals. Some professionals, such as Dental Hygienist 1, debrided the area by repeatedly executing single strokes in one direction. Other professionals, e.g., Dental Hygienist 2, maintained continuous contact between the instrument and the treated surface, and removed calculus by moving the instrument forward and backward along the surface.

One institution withdrew its consent to participate in the study shortly before the start of the measurements, limiting the number of participants. Due to the small number of measurements, the statistical power of this study is limited, and the results may not be generally valid. However, despite the limited number of measurements, the results allow estimating the magnitude and direction of the applied force, its rate of change, and the influence of profession and calculus hardness, and indicate that the force applied on hard artificial calculus is statistically significantly higher than on soft calculus, while dental hygienists and dentists apply similar forces. We suspect that the results would be similar with a higher number of measurements.

The data presented in this study can help to assess the clinical outcome of debridement with piezoelectric ultrasonic scalers and contributing factors in more detail and allow professionals to critically evaluate and eventually correct the force they apply and their work patterns.^13^

Furthermore, knowledge about the operation conditions during use is crucial for developing improved ultrasonic periodontal scalers. On the one hand, ultrasonic scalers must tolerate extreme operating conditions and different work patterns without major malfunction or damage. On the other hand, future research should investigate whether appropriate feedback to the operator, e.g., warning when the force exceeds a threshold, can help to reduce the force applied during debridement and consequentially improve the clinical results.

## Conclusion

The debridement force is correlated to the hardness of the calculus. Even trained and experienced professionals occasionally apply forces exceeding recommendations by the manufacturer and the literature, particularly on comparatively hard calculus.
